# Pathogenesis of Pregnancy-Related Complications

**DOI:** 10.3390/ijms24065584

**Published:** 2023-03-15

**Authors:** Ilona Hromadnikova

**Affiliations:** Department of Molecular Biology and Cell Pathology, Third Faculty of Medicine, Charles University, 100 00 Prague, Czech Republic; ilona.hromadnikova@lf3.cuni.cz; Tel.: +42-029-651-1336

In this special edition (closed on 31 October 2022), 4 reviews, 13 original papers, 1 communication, and 1 case report are published. These papers were published by scientific teams from 24 countries, including China, Tunisia, Canada, France, Serbia, Portugal, Spain, Chile, Singapore, Australia, USA, Mexico, Czech Republic, Germany, Sweden, Finland, United Kingdom, Norway, Poland, Greece, Japan, Italy, Switzerland, and Israel, which is a really great achievement.

In the review of Chen et al. [1], the authors summarize the role of galectins, multifunctional regulators of cellular biological processes involving innate and adaptive immune responses, in human reproduction, pregnancies with normal course of gestation and pregnancy-related disorders such as preeclampsia, gestational diabetes mellitus, fetal growth restriction and preterm birth.

The review of Benkhalifa et al. [2] was dedicated to the endometrium immunomodulation to prevent recurrent implantation failures and repeated pregnancy loss in women undergoing an in vitro fertilization programme. Various possible therapeutical options are reviewed together with a profiling of predictive biomarkers of implantation before embryo transfer.

The review of Vilotić et al. [3] provided a comprehensive overview on the role of IL-6 and IL-8, inflammatory cytokines, in cycling endometrium, the feto–maternal interface, establishment of pregnancy, parturition, and in the pathogenesis of pregnancy-related complications such as pregnancy loss, preeclampsia, gestational diabetes mellitus and infection/inflammation.

Ângelo-Dias et al. [4] performed a systematic review and meta-analysis assessing the association of B cells with idiopathic recurrent pregnancy loss. They highlighted a potential association between increased levels of endometrial B cells and idiopathic recurrent pregnancy loss.

Ortega et al. [5] demonstrated that pregnancy-induced chronic venous disease is associated with a proinflammatory environment characterized by altered serum levels of multiple inflammatory cytokines and chemokines in affected women and their newborns, which might have serious consequences for both.

Peñailillo et al. [6] described the potential communication between maternal mesenchymal stem cells derived from menstrual fluid and invading trophoblast cells during the implantation process. Reduced mesenchymal stem cell-induced trophoblast invasion was observed in patients with a history of preeclampsia.

Martinez-Fierro et al. [7] present interesting findings on the pathogenesis of preeclampsia, evaluating the effect of the administration of fibroblast growth factor type 2 on the placental expression of various genes involved in angiogenesis and apoptosis in an experimental murine model of preeclampsia. The administration of fibroblast growth factor type 2 reduced the effects generated by proteinuria and hypertension and also impacted the expression of studied genes.

Our research group [8] introduced a set of cardiovascular disease-associated microRNAs as potential biomarkers for the early identification of pregnancies at risk of later development of gestational diabetes mellitus. A first trimester screening of particular microRNAs allowed the authors to predict the later occurrence of gestational diabetes mellitus both irrespective of or with respect to the severity of the disease (group of all patients, group of patients on diet only, and group of patients on the combination of diet and therapy).

Czamara et al. [9] showed that children from pregnancies with an estimated first trimester risk based on fetal nuchal translucency thickness and abnormal maternal serum levels of first trimester routine biomarkers have a higher likelihood of adverse outcomes, even if initial testing of known genetic conditions is negative. In these children, congenital malformations of the circulatory system are more frequent. These children also have more copy number duplications. These cases should therefore be followed-up during pregnancy and after the birth.

Huang et al. [10] demonstrated that PEG2-induced pyroptosis affects the progression of endometriosis by changing the migration ability of pyroptotic cells and through the upregulation of HMGB1, E-cadherin, and vimentin. These findings might support the usage of anti-inflammatory drugs in patients with endometriosis.

Lynch et al. [11] reported that a deficiency in SLC2A3, a glucose transporter located on the maternal-facing apical trophoblast membrane, results in fetal hypoglycemia, reduced fetal development, and altered metabolic hormone concentrations in sheep.

Misan et al. [12] described the destabilization of the blood–brain barrier in pregnancies complicated by fetal growth restriction. Neurological disorders in newborns, including intraventricular hemorrhage, were associated with higher serum levels of NME1, nucleoside diphosphate kinase A, and the decreased placental expression of CLN4. Both biomarkers may be predictive of the appearance of intraventricular hemorrhage in newborns in FGR pregnancies.

Mavreli et al. [13] introduced miR-125a-3p as a promising early biomarker for prediction of spontaneous preterm birth. Mir-125a-3p was identified as a potential biomarker for the early prediction of spontaneous preterm birth using small RNA-seq and confirmed by qRT-PCR.

Szala-Poździej et al. [14] reported that some FCN2 gene promoter region polymorphisms that are associated with relatively low serum levels of ficolin-2 significantly increase the risk of very low birthweight in preterm neonates.

Kedziora et al. [15] studied gene expression signatures related to the diabetic placental pathology and concluded that fetal sex has a prominent effect on the placental transcriptome in diabetic pregnancies.

Kadife et al. [16] demonstrated that the expression of SLC38A4, a system A transporter controlling non-essential amino acid uptake and supply, is persistently low in placentas derived from pregnancies complicated with early preterm intrauterine growth restriction regardless of disease etiology.

Dymara-Konopka et al. [17] evaluated the serum levels of anti- and pro-angiogenic factors in pregnancies with preeclampsia and/or fetal growth restriction. They concluded that the angiogenic imbalance reflects placental disease regardless of its clinical manifestation in the mother.

Chighine et al. [18] presented a very rare case of a fatal respiratory failure two weeks after the delivery of a healthy newborn at home in a woman with normal course of gestation who suffered from a primary hyperparathyroidism secondary to a parathyroid carcinoma.

Finally, Sammar et al. [19] showed on in vitro models of preeclampsia that the overexpression of STOX1-A/B transcription gene, discovered in familial forms of preeclampsia, leads to the decreased expression of CD24, a mucin-like immunosuppressing glycoprotein, which was observed in syncytiothrophoblasts and cytotrophoblasts in early and preterm preeclampsia.
